# Protein Arginine Methyltransferase (PRMT) Inhibitors—AMI-1 and SAH Are Effective in Attenuating Rhabdomyosarcoma Growth and Proliferation in Cell Cultures

**DOI:** 10.3390/ijms22158023

**Published:** 2021-07-27

**Authors:** Joanna Janisiak, Patrycja Kopytko, Marta Tkacz, Dorota Rogińska, Magdalena Perużyńska, Bogusław Machaliński, Andrzej Pawlik, Maciej Tarnowski

**Affiliations:** 1 Department of Physiology Pomeranian Medical University, Powstańców Wielkopolskich 72, 70-111 Szczecin, Poland; joanna.bujak@onet.pl (J.J.); patrycja.kopytko@op.pl (P.K.); tkacz.mag@gmail.com (M.T.); pawand@poczta.onet.pl (A.P.); 2 Department of General Pathology, Pomeranian Medical University, 70-111 Szczecin, Poland; doroginska@gmail.com (D.R.); machalin@pum.edu.pl (B.M.); 3 Department of Experimental & Clinical Pharmacology, Pomeranian Medical University, 70-111 Szczecin, Poland; magdalena.peruzynska@pum.edu.pl

**Keywords:** epigenetics, RMS, methyltransferases, inhibitors

## Abstract

Rhabdomyosarcoma (RMS) is a malignant soft tissue cancer that develops mostly in children and young adults. With regard to histopathology, four rhabdomyosarcoma types are distinguishable: embryonal, alveolar, pleomorphic and spindle/sclerosing. Currently, increased amounts of evidence indicate that not only gene mutations, but also epigenetic modifications may be involved in the development of RMS. Epigenomic changes regulate the chromatin architecture and affect the interaction between DNA strands, histones and chromatin binding proteins, thus, are able to control gene expression. The main aim of the study was to assess the role of protein arginine methyltransferases (PRMT) in the cellular biology of rhabdomyosarcoma. In the study we used two pan-inhibitors of PRMT, called AMI-1 and SAH, and evaluated their effects on proliferation and apoptosis of RMS cells. We observed that AMI-1 and SAH reduce the invasive phenotype of rhabdomyosarcoma cells by decreasing their proliferation rate, cell viability and ability to form cell colonies. In addition, microarray analysis revealed that these inhibitors attenuate the activity of the PI3K-Akt signaling pathway and affect expression of genes related to it.

## 1. Introduction

Rhabdomyosarcoma (RMS) is a rare malignant neoplasm originating in the mesenchyme or neuroectoderm. Despite the low incidence (4.5 cases per million children), it is the most common sarcoma of soft tissues in children [1,2]. Due to the histopathological criteria are four main types of rhabdomyosarcoma: embryonal (EMRS), alveolar (ARMS), pleomorphic (PRMS), and spindle/sclerosing (ssRMS) [3]. Most diagnosed cases of rhabdomyosarcoma are isolated, sporadic cases, but there are genetic syndromes that favor the development of the disease, such as Beckwith–Widemann, Li Fraumeni, and Noonan syndrome [4]. Major genetic changes seen in RMS patients are translocations between chromosomes 1 and 13 and between chromosomes 2 and 13 [5]. Relatively recently, VGLL2/NCOA2/CITED2 and TFCP2/NCOA2 rearrangements as well as MYOD-1 point mutation were discovered in RMS patients [6]. Additionally, changes in genetic expression of IGF2, H19, CDKN1C, MYCN, MDM2 and PIK3CA are observed [7,8,9,10,11]. The molecular basis of rhabdomyosarcoma is not fully understood. Increasing evidence indicates that the development of RMS may involve not only mutations within the genome, but also epigenetic modifications [12,13]. The epigenome regulates chromatin architecture and influences the interactions between the DNA, histones and chromatin binding proteins, so any changes in the epigenome result in alterations in gene expression [14]. Additionally, modifications including methylation, acetylation, phosphorylation, sumoylation and glycosylation are reversible reactions, which makes them putative targets for anticancer therapies [15]. Previous studies on epigenomic pattern disturbances in rhabdomyosarcoma have focused mainly on two groups of epigenetic enzymes: DNA methyltransferases and histone deacetylases [13,16,17]. The aim of this study was to determine the effect of arginine methylation, and the enzymes involved in this process, on the biology of rhabdomyosarcoma cells.

Protein arginine methyltransferases (PRMTs) are a family of enzymes responsible for the methylation of arginine residues in histone tails, or non-histone proteins [18]. In the human body, there are known to be 9 different arginine methyltransferases, encoded by the PRMT1-PRMT9 genes [19]. Based on the end product of the reaction (when the methyl group is attached to the R residue), arginine methyltransferases have been divided into 3 classes: PRMT I, PRMT II and PRMT III. The first step of the reaction in all classes is the same and consists of the production of monomethylarginine (MMA) [20]. The next step of methylation is different for each class of enzymes. Among the class I arginine methyltransferases are PRMTs 1, 2, 3, 4, 6 and 8. These enzymes catalyse the formation of asymmetric dimethylated arginine (ADMA) [21]. Class II arginine methyltransferases include PRMT5 and PRMT9. These enzymes participate in the formation of symmetrically dimethylated arginine (SDMA) [22]. PRMT7 is the only enzyme belonging to the class III arginine methyltransferases group and it catalyses the formation of MMA [23]. A growing body of evidence shows that arginine methylation plays a key role in driving the appropriate expression of genes required during various stages of cell life. Abnormal PRMT levels may alter the activity of oncogenes and suppressor genes, and promote the modification of intracellular signalling pathways, promoting neoplastic transformation of cells [24,25]. In this study, we examined whether abnormal PRMT levels are present in the rhabdomyosarcoma and showed that inhibition of PRMT activity is effective in attenuation of RMS proliferation and growth.

## 2. Results

### 2.1. Rhabdomyosarcoma Cell Lines Show Increased Expression of Genes Encoding: PRMT1, PRMT4, PRMT5, PRMT6 and PRMT7

**Figure 1:**
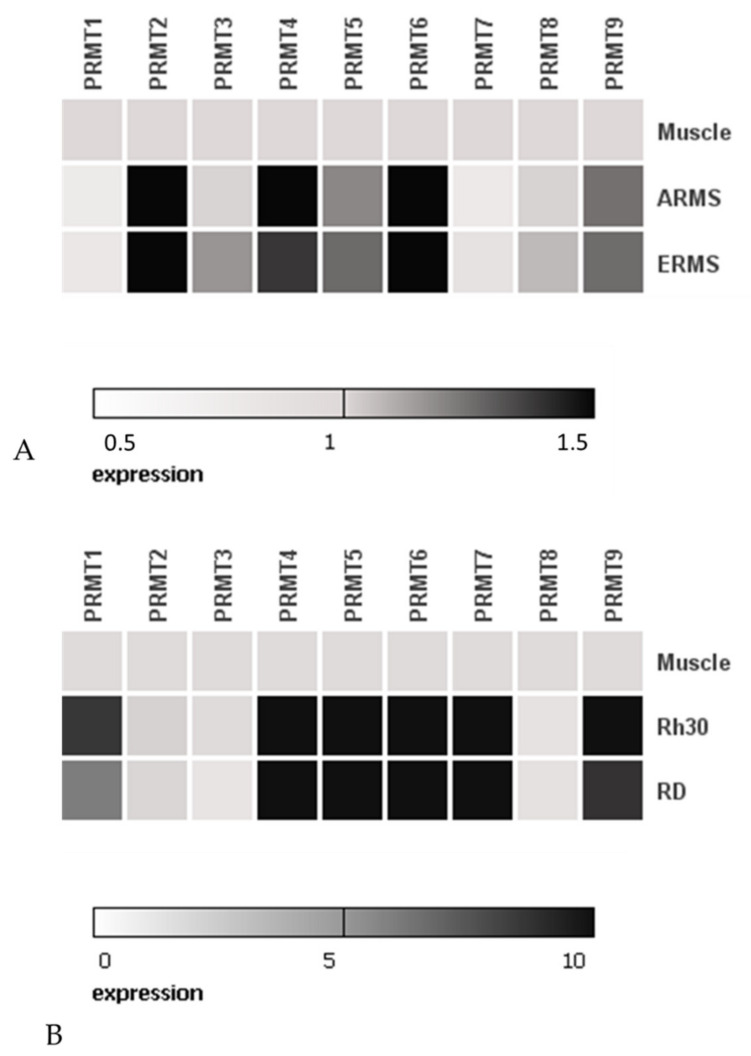
Heat map showing fold differences in PRMT1-PRMT9 expression. Panel (**A**) shows comparison of PRMTs expression between ARMS patients samples (*n* = 10), ERMS (*n* = 8) and normal muscle tissue samples (*n* = 3). Data were derived from GEO accession number GSE28511. Panel (**B**) shows PRMTs RQ-PCR expression in RMS cell lines and normal muscle sample. In Panel A and B normal skeletal muscle expression is equal to 1.

Increased expression of genes encoding PRMT has been detected in many neoplastic diseases, including in: leukaemia, melanoma, breast cancer and lung cancer [26,27,28]. In order to compare the expression levels of genes encoding arginine methyltransferases (PRMT 1-9), we performed real-time PCR in established human rhabdomyosarcoma cell lines: Rh30 and RD, and in normal skeletal muscle tissue. Rh30 cell line harbors PAX3-FOXO1 translocation and expresses fusion protein, is derived from bone marrow biopsy metastasis and possess alveolar histology, while RD cell line is of embryonal histology, translocation negative. We observed increased expression of 6 out of 9 tested arginine methyltransferases, specifically: PRMT1, PRMT4, PRMT5, PRMT6, PRMT7, PRMT8 and PRMT9 (Figure 1B). Additionally, using publicly available raw data from RMS tumors deposited in the Gene Expression Omnibus (GEO) (accession number GSE28511) and analysis software GEO2R, we evaluated PRMTs expression on 8 samples of ERMS, 10 samples of ARMS tumors and 3 normal skeletal muscle samples (Figure 1A). We noted that, although the number of samples in GEO is limited, expression of PRMT2, PRMT4-6 and PRMT9 is elevated in tumor samples.

### 2.2. Inhibition of PRMT Activity Reduces the Viability, Proliferation and Clonogenicity of Rh30 and RD Cells

**Figure 2:**
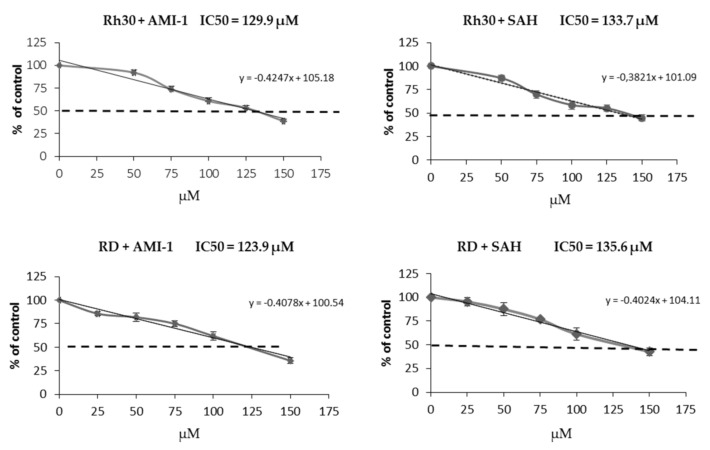
IC50 value was assessed for both of the RMS cell lines and for two inhibitors. The cells were cultured for 72 h in the presence of AMI-1 and SAH and then stained with WST-1. Viability was calculated accordingly to the formula: viability (% of vehicle control) = [(A_analyzed_ − A_blank_/A_control_ − A_blank_)] × 100%.

In order to reduce the activity of PRMT in RMS cells, we used two inhibitors of arginine methyltransferases with a broad spectrum of activity, AMI-1 (arginine methyltransferase inhibitor 1) and SAH (S-adenosyl-l-homocysteine). The anti-tumour nature of the selected inhibitors has been proven in lung, breast and prostate cancers, among others [29,30,31]. To assess the cytotoxicity of AMI-1 and SAH on rhabdomyosarcoma cells, we performed the WST-1 viability test, which allowed us to define the IC50 values of the tested substances in relation to RMS cells (Figure 2). Both PRMT inhibitors tested showed a similar degree of toxicity to RMS cells. The IC50 value of AMI-1 for the Rh30 line is 129.9 µM and 123.9 µM for the RD line, while for SAH it is 133.7 µM and 135.6 µM, respectively.

**Figure 3:**
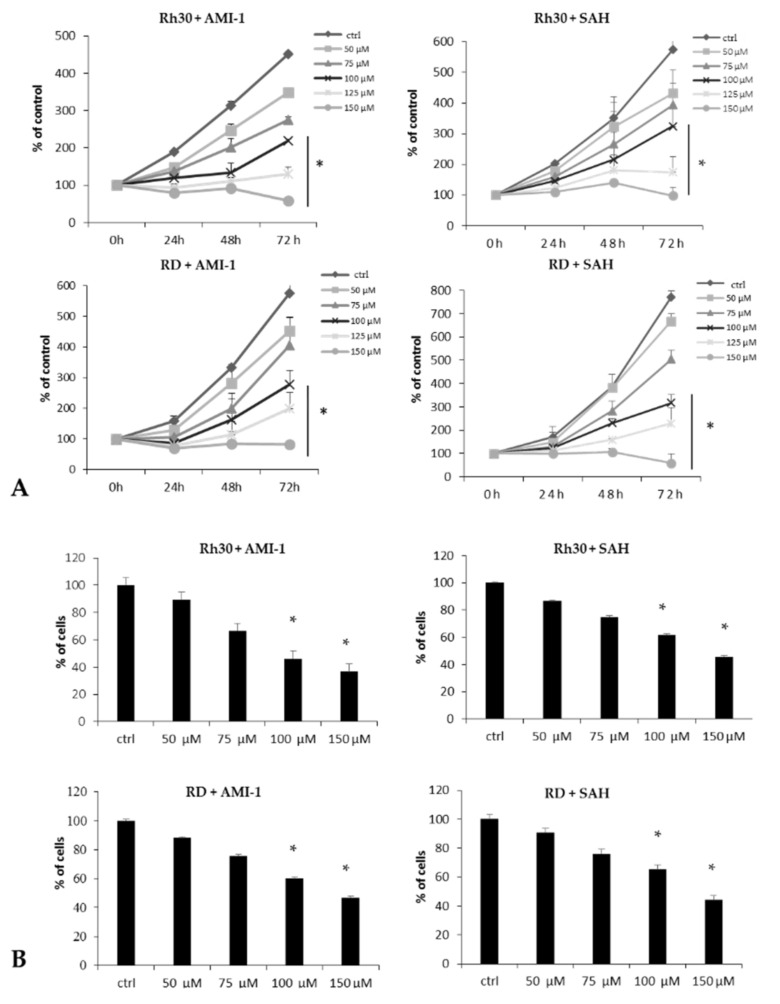
Panel (**A**): Evaluation of the proliferation rate of RMS cells in the presence of a concentration gradient of AMI-1 or SAH (50, 75, 100, 125, 150 µM). The proliferation index was calculated relative to the number of cells at the start of the experiment (0 h), which was 100%. The results from 3 independent experiments are presented. Panel (**B**): Assessment of RMS cell clonogenicity in the presence of a concentration gradient of AMI-1 or SAH (50, 75, 100, 150 µM). 100% is the number of clones produced by ctrl (untreated) cells. Unstained cell colonies were counted using a light microscope 21 days after setting up the experiment. * *p* < 0.05 calculated vs. ctrl (untreated) cells.

Next, we examined the inhibitory effect of AMI-1 and SAH on the growth kinetics of RMS cells. As shown in Figure 3A, we saw a statistically significant decrease in Rh30 and RD cell proliferation after exposure to AMI-1 or SAH for 24, 48 and 72 h (adhesion dependent growth). Moreover, the tested PRMT inhibitors inhibited colony formation on soft agar (adhesion independent growth), as shown in Figure 3B.

**Figure 4:**
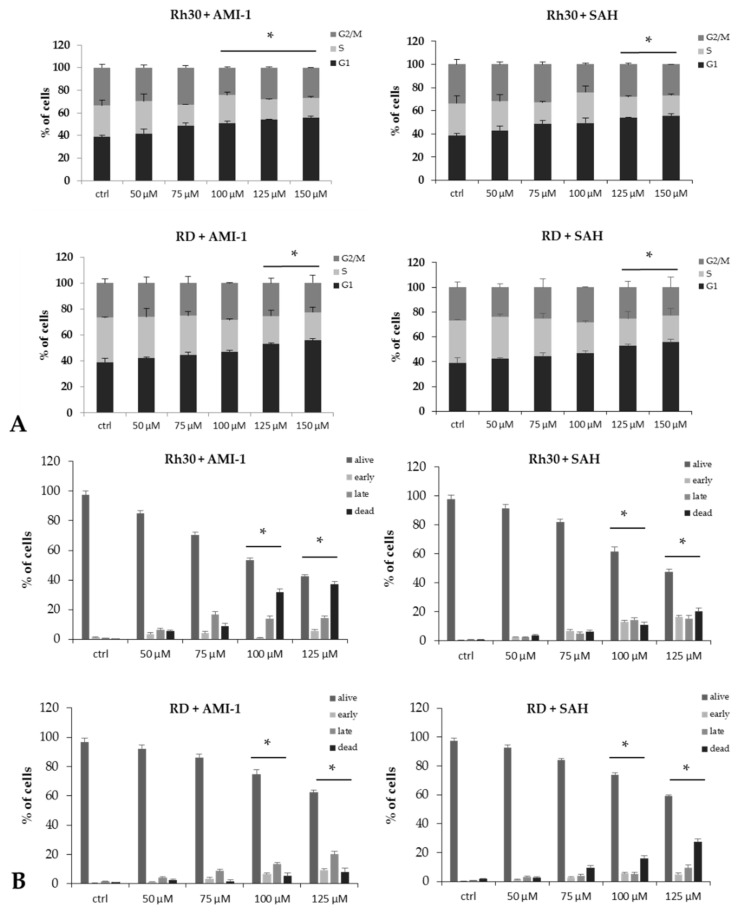
Panel (**A**). Cell cycle course for RMS cells cultured in a concentration gradient of AMI-1 or SAH (50, 75, 100, 125, 150 µM). * Statistically significant difference for the G1 phase of the cell cycle (*p* < 0.05) compared to the G1 phase of the control group (ctrl). Panel (**B**). Assessment of apoptosis in RMS cells grown with a concentration gradient of AMI-1 or SAH (50, 75, 100, 125 µM). * Statistically significant difference for the number of cells compared to the number of control (untreated) cells. * *p* < 0.05.

The observed decrease in the rate of proliferation and the decreased ability to form colonies by RMS cells after exposure to AMI-1 and SAH may result from alterations in the cell cycle. We examined the influence of the tested PRMTs inhibitors on the course of the cell cycle using flow cytometry and Vybrant DyeCycle Orange Stain reagent. As shown in Figure 4A, AMI-1 and SAH increase the percentage of RMS cells remaining in the G1 phase of the cell cycle. The reduction of PRMT activity by specific inhibitors such as AMI-1 or SAH may induce apoptosis of neoplastic cells [32,33]. We used annexin V and propidium iodide staining to test the pro-apoptotic properties of AMI-1 and SAH in Rh30 and RD cells. Cytometric analysis showed induction of apoptosis in both cell lines after the administration of AMI-1 and SAH at a dose of ≥100 µM (Figure 4B).

### 2.3. Changes in the Gene Expression Profile of RMS Cells after Exposure to AMI-1 and SAH

**Figure 5:**
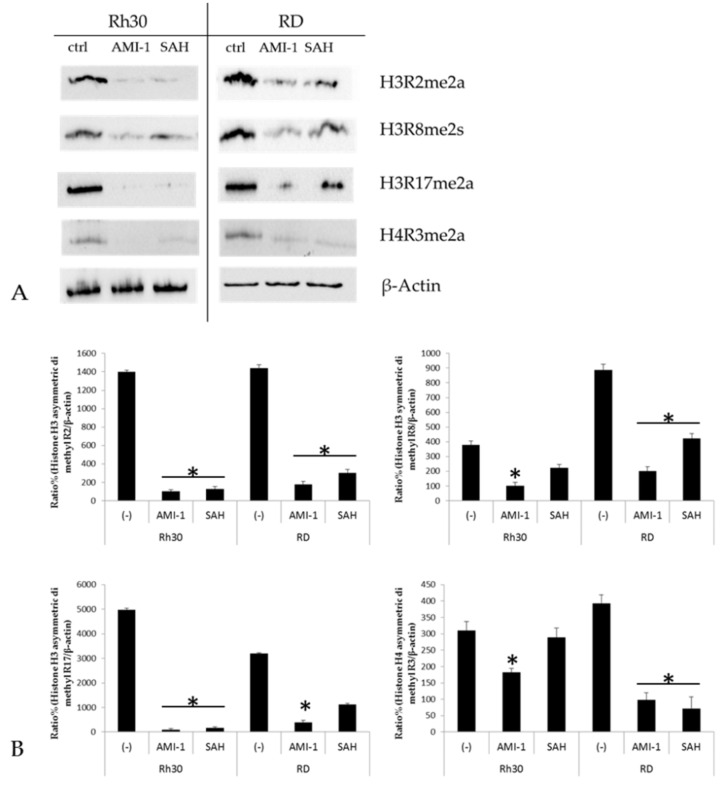
Panel (**A**). Western blot analysis of PRMTs activity blockage by pan-inhibitors AMI-1 and SAH. Rh30 and RD cell lines were treated with the inhibitors 100 µM for 48 h and methylation at H3R2me2a, H3R8me2s, H3R17me2a, H4R3me2a was evaluated by arginine methylation specific antibodies. Panel (**B**) shows the quantitative Western blot analysis, expressed as the ratio of methylated histone protein to loading control (β-Actin). * *p* < 0.05.

The mechanism of action of AMI-1 and SAH is based on blocking the activity of arginine methyltransferases, meaning that these substances are able to significantly modify the epigenome [33,34]. Firstly, we noted that the inhibitors (which are pan inhibitors of PRMTs) significantly reduced the level of methylation in selected histone marks (H3R2me2a, H3R8me2s, H3R17me2a, H4R3me2a). The weakest level of arginine methylation was present in H4R3me2a, which is methylated mostly by PRMT1 (Figure 5); nonetheless, AMI-1 effectively inhibited this methylation.

**Figure 6:**
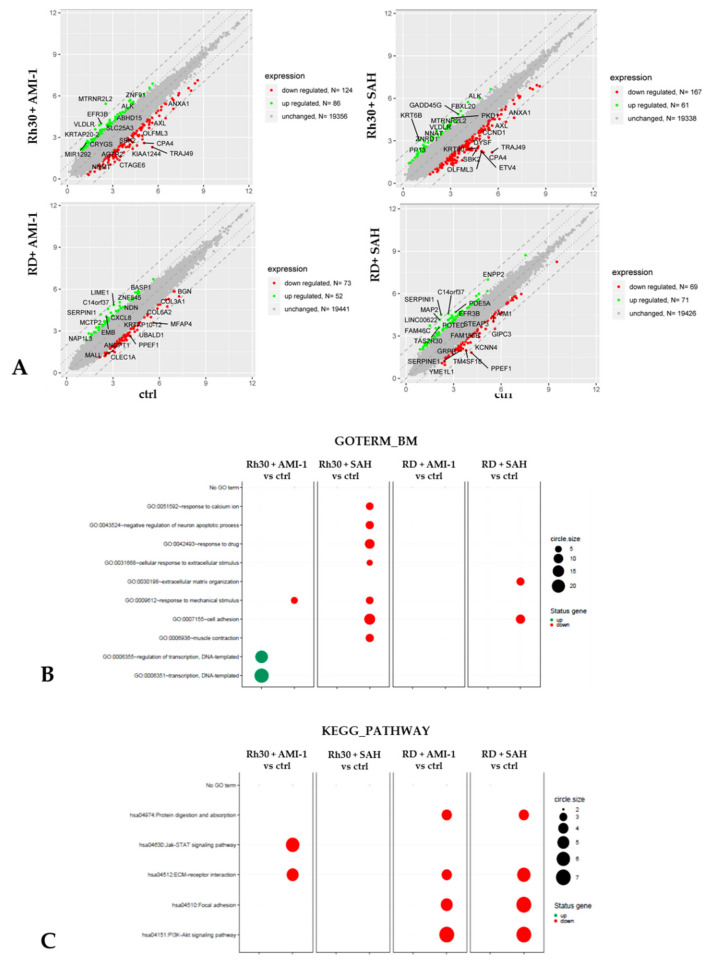
Panel (**A**). Scatter plot of microarray data. Data are presented in a scatter plot to visualize changes in gene expression between control group and AMI-1 and SAH (100 µM) treated RMS cells. Each gene is marked with a dot. Genes marked in green increased their expression at least 2-fold, while genes with at least 2-fold decrease in expression compared to controls are represented by red dots. The symbols of the genes with the largest expression differences are also shown in the graph. Panel (**B**). List of functions of biological processes modified by AMI-1 and SAH in cells of Rh30 and RD line, obtained on the basis of gene ontology classification (GO). Panel (**C**). List of functions of biological processes modified by AMI-1 and SAH in Rh30 and RD cells, obtained on the basis of the KEGG set. The size of each dot represents number of genes involved; fold difference >2 or <−2; *p* < 0.05, *FDR*-adjusted *p*-value was less than 0.2.

To identify the molecular changes in RMS cells after exposure to the tested PRMT inhibitors, we conducted a detailed comparison between the transcription profiles of Rh30 and RD cells cultured in the presence of AMI-1 and SAH and control cells. Quantitative analysis of RNA microarrays allowed for the identification of gene transcripts, which showed at least a 2-fold difference in expression levels between the analysed groups (Figure 6A). In the group of Rh30 cells cultured in the presence of AMI-1, we identified 210 genes whose expression differed in comparison to the cells of the control group. Among them, 86 genes were overexpressed by a factor of 2–7.40, while in 124 genes we noted a decrease in expression by a factor of 2–9.98. In the case of RD cells grown in the presence of AMI-1, we identified 125 genes with altered expression, of which 52 were overexpressed and 73 were negatively regulated in relation to the cells of the control group. In Rh30 cells incubated in the presence of SAH, we detected 228 genes that were differentially expressed relative to the control cells. In 61 genes there was a 2–4.09-fold increase in expression, while 167 genes showed a 2–10.78-fold decrease in expression, relative to the control cells. RD cells grown in the presence of SAH also showed altered gene expression. We identified 71 positively regulated genes and 69 genes with reduced expression compared to cells in the control group.

Next, the identified altered genes were tested against biological pathways using the Gene Ontology (GO) classification and the Kyoto Encyclopaedia of Genes and Genomes (KEGG). As shown in Figure 6B,C, functional analysis revealed a number of biological processes that were significantly negatively regulated in the group of RMS cells incubated in the presence of AMI-1 and SAH. RD cells cultured in the presence of AMI-1 and SAH showed reduced activity of the PI3K-Akt signalling pathway (hsa04151), while Rh30 cells incubated with AMI-1 had reduced JAK-STAT signalling (hsa04974). Both signal transduction pathways are over-activated in neoplastic diseases, and the weakening of their functions leads to a decrease in metabolism, proliferation, growth and migration of transformed cells [35,36]. These results seem to confirm the anti-tumour nature of the tested inhibitors. We also noted that application of AMI-1 and SAH did not affect PRMTs expression (data not shown). Another important function of the investigated PRMT inhibitors in the RD lineage is the negative regulation of the adhesion process (hsa04510, GO: 0007155), which is an important factor enabling cell survival. Oligonucleotide microarray analysis showed a positive regulation of DNA transcription (GO: 0006355, GO: 0006351) in Rh30 cells in the presence of AMI-1.

**Figure 7:**
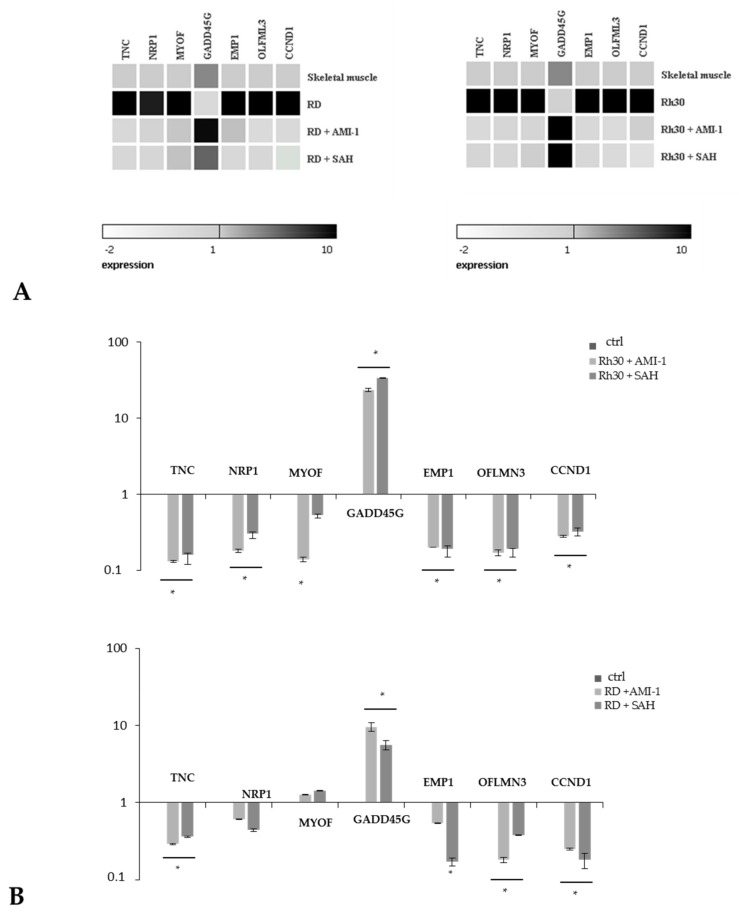
Panel (**A**). Heat maps of RQ-PCR results showing expression of genes selected by the microarray analysis in RMS cells. The activity of selected genes in RMS cells, normal skeletal muscle tissue and RD cells incubated in the presence of AMI-1 and SAH (100 µM) was compared. Panel (**B**). Expression of genes selected on the basis of oligonucleotide microarray analysis in RMS cells. The activity of selected genes in untreated RMS (ctrl) cells and RMS cells incubated in the presence of AMI-1 and SAH (100 µM) was compared. The relative expression of the genes in cells of the ctrl group is 1. * *p* < 0.05.

Based on the results obtained from the analysis of expression microarrays, we selected 7 genes whose expression changed in all combinations of the experiment. These were TNC, NRP1, MYOF, GADD45G, EMP1, OFLMN3 and CCND1. We confirmed the results obtained during the microarray analysis using RQ-PCR. We have shown that established Rh30 and RD sarcoma cell lines cultured in the presence of the tested PRMT inhibitors have decreased expression of TNC, NRP1, MYOF, EMP1, OLFML3 and CCND1 and increased expression of the GADD45G gene (Figure 7A,B). Additionally, we compared the expression of the above-mentioned genes in Rh30 and RD cells, cultured in the presence of AMI-1 and SAH, and in normal skeletal muscle. The results obtained prove that the genes TNC, NRP1, MYOF, EMP1, OFLMN3 and CCND1 are overexpressed in the RMS cells, while the GADD45G gene has a reduced expression compared to normal muscle tissue. Culturing Rh30 and RD cells in the presence of AMI-1 and SAH restored the expression of the selected genes close to normal levels, such as those seen in normal skeletal muscle (Figure 7A).

### 2.4. AMI-1 and SAH Decrease the Levels of Cyclin D1 and Bcl-xL and Increase the Level of GADD45G Protein by Negatively Regulating the PI3K-Akt Signalling Pathway

**Figure 8:**
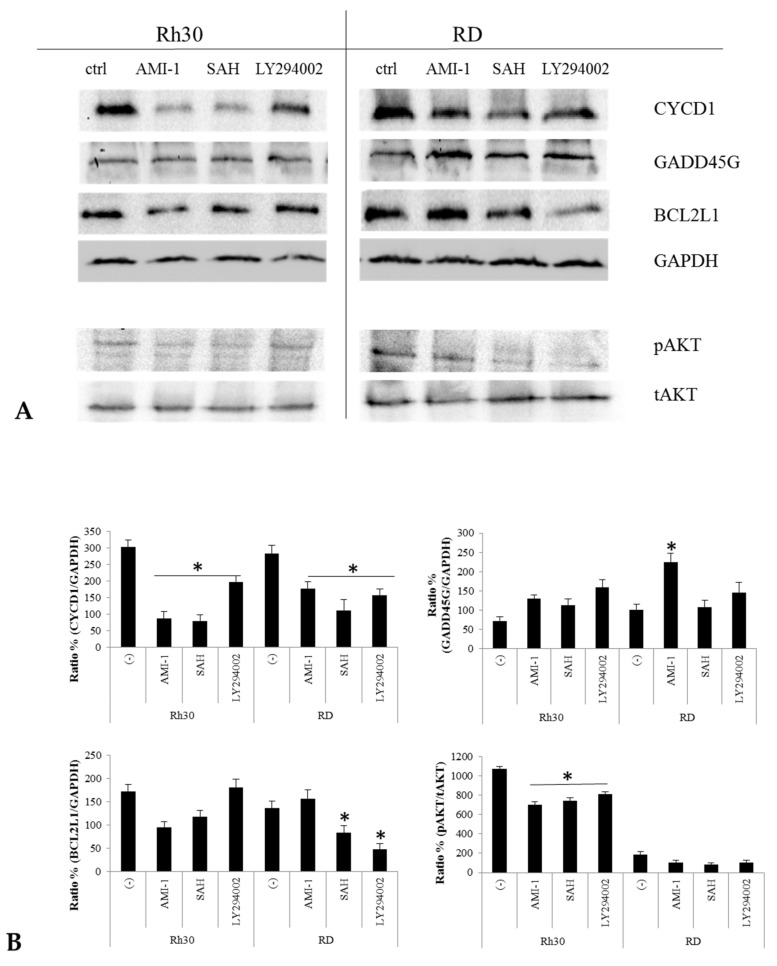
Effect of the inhibition by AMI-1, SAH or LY294002 on RMS cell culture. Rh30 and RD cells (panel **A**) were untreated or treated with AMI-1 (100 µM), SAH (100 µM) or LY294002 (1.4 µM) for 48 h. Phosphorylation was assessed by Western blot. Panel (**B**) shows the quantitative Western blot analysis, expressed as the ratio of phosphorylated protein to total protein (GAPDH or tAKT). * *p* < 0.05.

Functional analysis based on the KEGG showed that culturing RMS cells in the presence of AMI-1 and SAH led to a negative regulation of the seven genes responsible for the activity of the PI3K-Akt signalling pathway (CCND1, COL1A1, COL6A1, COL6A2, IL7R, SPP1, THBS2). The excessive activation of the PI3K-Akt pathway is one of the most frequently altered signal transduction pathways in cancer patients [35,36]. Reducing the activity of the PI3K-Akt signalling pathway results in reduced proliferation of neoplastic cells and promotes their apoptosis [35,36], among other effects. To check whether the tested PRMT inhibitors negatively regulate the PI3K-Akt signalling pathway, we performed a Western blot analysis. We used the potent PI3K-Akt inhibitor, LY294002, as a control. Additionally, we checked the levels of cyclin D1, Bcl-xL and GADD45G proteins. As shown in Figure 8, reducing the activity of the PI3K-Akt signalling pathway with AMI-1, SAH or LY294002 leads to a decrease in the levels of cyclin D1 and Bcl-xL protein, and an increase in the level of GADD45G protein.

## 3. Discussion

Arginine methyltransferases significantly affect the transcriptional activity of chromatin, meaning that their dysregulation may lead to the development of pathological conditions. PRMTs, acting as activators or repressors of transcription, may interfere with basic cellular processes, such as proliferation, differentiation, migration or apoptosis [37,38,39,40]. The influence of an incorrect level of PRMT on the pathogenesis of neoplastic diseases has been proven in many cancers, such as lung, prostate, colon, mammary gland and melanoma [41,42,43,44]. Increased PRMT levels promote disease progression; therefore, PRMTs have become a promising therapeutic target. In this study, we compared the expression of PRMT1-PRMT9 genes in normal muscle tissue and in Rh30 and RD RMS cell lines. We detected the overexpression of 6 out of 9 tested genes, specifically, PRMT1, PRMT4, PRMT5, PRMT6, PRMT7 and PRMT9. Overexpression of these enzymes in RMS cells is known to promote the development of neoplastic disease and correlates with greater invasiveness of transformed cells. Methyltransferases 1, 4 and 6 promote the uncontrolled proliferation of neoplastic cells by suppressing important cell cycle inhibitors and activating Akt kinase and deregulation of β-actin [27,37,42,43]. Increased PRMT5 levels disturb the course of the cell cycle and abolish the process of programmed cell death [44,45,46]. However, the increased concentration of PRMT7 promotes the spread of the disease by promoting distant metastases [47,48]. To evaluate the effect of increased PRMT levels on the biology of RMS cells, we used two inhibitors of arginine methyltransferases with a broad spectrum of activity: AMI-1 and SAH. Both inhibit the proliferation of various neoplastic cells in vitro and in vivo [49]. Thus, it appears that PRMTs are pivotal enzymes that modulate expression of genes responsible for cell proliferation and growth, by methylation of arginine residues in the histone tails. Zhang et al. proved that AMI-1 attenuates growth kinetics of colorectal cancer cells, and the observed anti-proliferative effect is directly proportional to the increasing concentration of AMI-1 [29]. Our results also indicate that AMI-1 and SAH reduce the rate of RMS cell proliferation in a way that is directly proportional to the increasing doses of the tested PRMT inhibitors (Figure 3A). The decrease in the growth kinetics of RMS cells after the use of AMI-1 and SAH may be due to a decrease in the activity of the PI3K-Akt signalling pathway, as confirmed by a functional analysis based on the results obtained from the analysis of expression microarrays (Figure 6B,C). The PI3K-Akt pathway undergoes increased activation in many types of cancer and positively influences the process of proliferation of transformed cells [50,51]. Over-activation of the PI3K-Akt pathway is one of the most frequently altered signal transduction pathways in rhabdomyosarcoma, affected in approximately 59% of ARMS and 29% of ERMS cases, making it a potential therapeutic target [52]. Increased activity of the PI3K-Akt signalling pathway may promote cell proliferation through excessive accumulation of cyclin D1. Akt positively regulates the level of cyclin D1 by inactivating the kinase of glycogen synthase 3-beta (GSK3β). This results in reduced phosphorylation of cyclin D1 at Thr286, inhibition of nuclear export, and inhibition of cytoplasmic degradation of the proteasomal cyclin D1 [50]. In the present study, we demonstrated that reducing the activity of the PI3K-Akt signalling pathway using AMI-1 and SAH results in decreased levels of cyclin D1 (Figure 8). As a result of this, RMS cells stall in the G1 phase of the cell cycle (Figure 4A).

Blocking PRMT activity in many tumours has proven to be an effective way to induce apoptosis in transformed cells of osteosarcoma, colorectal cancer, lung cancer and melanoma [29,49,53,54]. We have shown that AMI-1 and SAH significantly affect the level of histone arginine methylation at H3R2me2a, H3R8me2s, H3R17me2a, H4R3me2a (Figure 5) In our study, we observed that the use of AMI-1 and SAH in RMS cells also resulted in a statistically significant increase in apoptosis (Figure 3B). Due to the wide spectrum of activity of the tested substances, the observed pro-apoptotic effect may result from the regulation of various processes involved in programmed cell death. Thanks to the results obtained during the analysis of oligonucleotide microarrays, we identified several phenomena affected by AMI-1 and SAH that can induce apoptosis of RMS cells. In particular, the use of AMI-1 or SAH lowered the expression of the BCL2L1 gene and increased the expression of the GADD45G gene. The intrinsic pathway of apoptosis is regulated by pro- and anti-apoptotic proteins, and the proportion between them determines the sensitivity of cells to stimuli that may cause cell death [55]. The BCL2L1 gene belongs to the BCL2 protein family, and the Bcl-xL protein encoded by it is classified as an apoptosis inhibitor. The mode of action of the Bcl-xL protein is based on blocking voltage-dependent anion channels (VDAC), which prevents the release of cytochrome c1 [56]. In our study, we observed a decrease in the expression of the BCL2L1 gene after the application of AMI-1 and SAH in Rh30 cells, which sensitised the cells to the apoptosis process. Leverrier et al. demonstrated that the PI3K-Akt signalling pathway targets members of the Bcl-2 family [57]. The PI3K-Akt pathway stimulates the expression of anti-apoptotic proteins, such as Bcl-2, Bcl-xL and Mcl-1, by activating NF-kB [58]. The observed pro-apoptotic effect in RMS cells after the use of AMI-1 and SAH can also be explained by the increased expression of the GADD45G gene (Figure 7A,B). Under physiological conditions, the GADD45G gene is expressed in all normal tissues. The main task of the protein encoded by GADD45G is to induce a stress shock response, which leads to cell apoptosis [59]. Transcriptional silencing of the gene and hypermethylation of its promoter region have been observed in many neoplastic tissues [60,61]. The analysis of the transcriptome showed that also in the case of rhabdomyosarcoma, the expression of the GADD45G gene is reduced. There are indications that PRMT1 may be indirectly involved in the regulation of the gene activity. Among other processes, the enzyme participates in the attachment of a methyl group to the BRCA1 protein. Blockade of this methylation reaction facilitates the recruitment of BRCA1 to the promoter of the GADD45G gene and promotes its further activation [62]. In this study, we showed that the use of AMI-1 and SAH in RMS cells restored the expression of the GADD45G gene mRNA to a level comparable to normal muscle tissue (Figure 7A) and increased the protein concentration in the tested cells (Figure 8). The increase in GADD45G gene expression in RMS cells after the use of AMI-1 and SAH may be due to a decrease in the activity of the PI3K-Akt signalling pathway. The relationship between the activity of the PI3K-Akt signalling pathway and the expression of genes from the GADD45 family in sarcoma cells (including RMS cells), has been proven by Zhu et al. [63]. Blocking the activity of the PI3K-Akt signalling pathway in sarcoma cells leads to an increase in GADD45G gene expression, halts the cell cycle and facilitates apoptosis [63].

## 4. Materials and Methods

### 4.1. Reagents

AMI-1 (arginine methyltransferase inhibitor 1) and SAH (S-adenosyl-l-homocysteine) inhibitors were purchased from Sigma-Aldrich (St. Louis, MO, USA). The inhibitors were dissolved in water.

### 4.2. Cell Lines

We used human RMS cell lines RH30 (ARMS) and RD (ERMS) (purchased in ATCC). RMS cells used for experiments were cultured in Roswell Park Memorial Institute medium (RPMI) 1640 and Dulbecco’s Modified Eagle’s Medium (DMEM) (both Sigma, St. Louis, MO, USA), supplemented with 100 IU/mL penicillin and 10 µg/mL streptomycin (Life Technologies, Inc., Grand Island, NY, USA) in the presence of 10% heat-inactivated fetal bovine serum (FBS, Life Technologies). The cells were cultured in a humidified atmosphere at 37 °C in 5% CO_2_ at an initial cell density of 2.5 × 10^4^ cells/flask (Corning, Cambridge, MA, USA) and the media were changed every 48 h.

### 4.3. Viability Test

The cell viability was evaluated using the Cell Proliferation Reagent WST-1 assay (Sigma Aldrich). The RMS cells were seeded in 96-well plate at a density of 2 × 10^3^ cells/well. The cells were cultured in 100 uL medium with respective AMI-1 and SAH (25, 50, 75, 100, 150 μM; Sigma-Aldrich) inhibitors. Cells cultured in 10% FBS served as controls. After 72 h of culture 10 µL WST-1 reagent was added and cells were incubated for 30 min. Afterwards, absorbance was measured using a spectrophotometric microplate reader (Infinite 200 Pro, Tecan) at 450 nm (with 620 nm background correction). Results were normalized to the control, non-stimulated cells. Calculation of cell viability: number of viable cells = [(test-blank)/(control-blank)] × 100%. The experiment was repeated three times in triplicates.

### 4.4. Proliferation Test

The RH30 and RD cells were plated on a 24-well plate at an initial density of 7 × 10^5^ per well in the presence or absence of AMI-1 and SAH (25, 50, 75, 100, 150 μM; Sigma-Aldrich) inhibitors. Proliferation of cells cultured in 10% FBS served as control. The number of cells was calculated at the time points: 24, 48 and 72 h. At each time point the cells were trypsinized in order to count them using Navios flow cytometer (Beckman Coulter, Inc., Brea, CA, USA). Every test was performed in duplicate. The experiment was repeated three times.

### 4.5. Colony Formation Assay

Cells were incubated with or without AMI-1 and SAH (50, 75, 100, 150 μM; Sigma-Aldrich) for 72 h, collected, counted, mixed in 0.35% Top Agar (in RPMI 1640 medium supplemented with 10% fetal bovine serum) and plated at 1.25 × 10^3^ cells/well onto 24-well plates containing a solidified bottom layer (0.5% Base Agar in the same growth medium). Every three days colonies were fed with 250 μL/well culture medium with 10% FBS with or without AMI-1 and SAH (50, 75, 100, 150 μM). After 21 days, unstained colonies were counted and photographed. The experiment was repeated three times.

### 4.6. Cell Cycle Analysis

The cells were incubated with or without AMI-1 and SAH (50, 75, 100, 150 μM; Sigma-Aldrich). After 48 h, the cells were collected, washed with PBS, centrifuged at 1200 rpm for 10 min and resuspended in 1 mL RPMI 1640 medium supplemented with 10% fetal bovine serum at a concentration of 10^6^ cells/mL. Then, 2 μL of Vybrant DyeCycle Orange Stain (Invitrogen, Waltham, MA, USA) was added to the cells and gently vortex. Samples were kept at 37 °C for 30 min in the dark and were analyzed by flow cytometer (Navios, Beckman Coulter) and ModFit LT 5.0 software. The experiment was repeated three times.

### 4.7. Annexin V/PI Assays for Apoptosis

For Annexin V/PI assays, cells were stained with Annexin V–FITC and PI, and evaluated for apoptosis by flow cytometry according to the manufacturer’s protocol (BD PharMingen, San Diego, CA, USA). Cells were seeded on 6-well plate at density 2.5 × 10^5^ cells/well and cultured in the presence or absence of AMI-1 or SAH (50, 75, 100, 125, 150 µM) for 72 h. Next, cells were washed twice with phosphate-buffered saline (PBS), and stained with 5 μL of Annexin V–FITC and 10 μL of PI (5 μg/mL) in 1× binding buffer (10 mM HEPES, pH 7.4, 140 mM NaOH, 2.5 mM CaCl_2_) for 15 min at room temperature in the dark. The apoptotic cells were determined using a Navios flow cytometer (Beckman Coulter). Viable cells were represented as Annexin V−/PI−, early apoptotic cells as Annexin V+/PI−, late apoptotic cells as Annexin V+/PI+, and necrotic cells as Annexin V−/PI+), all were quantified as a percentage of the gated population. The experiment was repeated three times.

### 4.8. Oligonucleotide Microarrays

The material for the evaluation of global gene expression using microarrays was RNA, which was isolated from Rh30 and RD cells cultured in the presence of tested PRMT inhibitors (100 µM), cells constituting the control group were cultured in standard medium supplemented with 10% FBS. Three independent experiments were performed. To minimize the effect of individual differences between the obtained samples, material derived from the 3 isolated RNAs within each group was combined. The sense-stranded cDNA was generated from RNA using the GeneChip™ WT PLUS Reagent Kit (Thermo Fisher Scientific, Waltham, MA, USA), fragmented and labeled with the GeneChip WT Terminal Labeling Kit (Thermo Fisher Scientific), and then hybridized with GeneChip™ Human Gene 2.1 ST Array Strip. Hybridization and subsequent fluidization and scanning steps were performed with the Affymetrix GeneAtlas™ system. Data readout including measurement of the fluorescence intensity of phycoerythrin was performed using a visualization station (Affymetrix Gene Atlas ™ Imaging Station). Then, the CEL directory was created as the output file for bioinformatics analysis. CEL files were imported into the Bioconductor application. Background correction, normalization and summary of raw results were developed using the RMA algorithm. The obtained results were combined with a descriptive library to give a complete list of genes. The selection of results between the tested samples was made after at least two-fold changes in gene expression compared to the control samples.

### 4.9. Real-Time Quantitative Reverse Transcription PCR (RQ-PCR)

Total RNA was isolated from cells treated with AMI-1 or SAH and from cell controls with the RNeasy Kit (Qiagen, Dusseldorf, Germany). The RNA was reverse-transcribed with MultiScribe reverse transcriptase and oligo-dT primers (Applied Biosystems, Foster City, CA, USA). Quantitative assessment of mRNA levels was performed by real-time RT-PCR (RQ-PCR) on an ABI 7500 instrument with Power SYBR Green PCR Master Mix reagent. Real-time conditions were as follows: 95 °C (15 s), 40 cycles at 95 °C (15 s), and 60 °C (1 min). According to melting point analysis, only one PCR product was amplified under these conditions. The relative quantity of a target, normalized to the endogenous control β-2 microglobulin gene and relative to a calibrator, is expressed as 2^−∆∆Ct^ (fold difference), where Ct is the threshold cycle, ∆Ct = (Ct of target genes) − (Ct of endogenous control gene, β-2 microglobulin), and ∆∆Ct = (∆Ct of samples for target gene) − (∆Ct of calibrator for the target gene). The expression of following genes were analyzed: PRMT1-9, TNC, NRP1, MYOF, GADD45G, EMP1, OLFML3, CYCD1. Primer sequences are available upon request.

### 4.10. Phosphorylation of Intracellular Pathway Proteins and Western Blotting

Western blots were performed on extracts prepared from RMS cell lines (3 × 10^6^ cells) that were kept in RPMI or DMEM medium containing low levels of bovine serum albumin (BSA, 0,5%) to render the cells quiescent. The cells were divided and stimulated with optimal doses of AMI-1 (100 µM), SAH (100 µM) and LY294002 (1.4 µM) for 48 h at 37 °C and then lysed (for 10 min) on ice in RIPA lysing buffer (Santa Cruz Biotech, Santa Cruz, CA, USA), containing protease and phosphatase inhibitors (Roche). Subsequently, the extracted proteins were separated by either 12% sodium dodecyl sulfate-polyacrylamide gel electrophoresis (SDS-PAGE) and the fractionated proteins were transferred to a nitrocellulose membrane (BioRad, Hercules, CA, USA). Phosphorylation of the intracellular kinases and expression of proteins was detected using commercial phospho-specific mAbAKT (Ser473) (Cell Signaling, Danvers, MA, USA) and anti-GADD45G ab196774, (abcam, Cambridge, UK), anti-BCL2L1 AV30475 (Sigma-Aldrich), anti-Cyclin D1 ab134175, (abcam) with horseradish peroxidase (HRP)-conjugated goat IgG as a secondary antibody (Santa Cruz Biotech). Equal loading in the lanes was evaluated by stripping the blots and reprobing with tAKT clone no. 9272 (Cell Signaling). The membranes were developed with an enhanced chemiluminescence (ECL) reagent (GE Healthcare), dried and visualized by Chemidoc transilluminator (BioRad). For histone methylation analysis the stimulated cells were incubated with 200 µL of TEB buffer (PBS supplemented with 0.5% Triton X-100, 2 mM PMSF and 0.02% NaN3) for 10 min on ice with gentle stirring. After centrifugation the pellet was resuspended in 200 µL of extraction buffer (0.5N HCl and 10% glycerol), incubated on ice for 30 min and centrifuged at 12,000 rpm for 5 min at 4 °C. Supernatant were transferred to a new tube with 150 µL of acetone and incubated at −20 °C overnight. The membranes were incubated with following antibodies: histone H3 asymmetric di methyl R2 (Abcam, ab175007), histone H3 symmetric di methyl R8 (Abcam, ab130740), histone H3 asymmetric di methyl R17 (Abcam, ab8284), histone H4 asymmetric di methyl R3 (Abcam, ab194683) and β-Actin (Abcam).

### 4.11. Statistical Analysis

All results are presented as mean ± standard error of the mean (SEM). Statistical analysis of the data was performed using the nonparametric Mann-Whitney test or Student *t*-test, with *p* < 0.05 considered significant.

## 5. Conclusions

Overall, our study showed that PRMTs are overexpressed in RMS, and we proved that the inhibition of arginine methyltransferases with broad-spectrum PRMT inhibitors, such as AMI-1 or SAH, has a strong anti-tumour effect on RMS cells. PRMT inhibitors reduce the invasive nature of RMS cells by reducing their proliferation, arresting the cell cycle and inducing apoptosis.

## Data Availability

The data presented in this study are available on request from the corresponding author.

## Abbreviations

RMS	rhabdomyosarcoma
ARMS	alveolar RMS
ERMS	embryonal RMS
PRMT	protein arginine methyltransferase
AMI-1	arginine methyltransferase inhibitor
SAH	S-adenosyl-l-homocysteine
